# Preclinical EIS Study of the Inflammatory Response Evolution of Pure Titanium Implant in Hank’s Biological Solution

**DOI:** 10.3390/molecules28124837

**Published:** 2023-06-18

**Authors:** Lidia Benea, Iulian Bounegru, Alexandra Forray, Elena Roxana Axente, Daniela Laura Buruiana

**Affiliations:** 1 Competences Center: Interfaces-Tribocorrosion-Electrochemical Systems, Dunarea de Jos University of Galati, 47 Domnească Street, RO-800008 Galati, Romania; elena.axente@ugal.ro (E.R.A.); daniela.buruiana@ugal.ro (D.L.B.); 2 Military Medicine Institute, Street Institutul Medico-Militar 3-5, RO-010919 Bucharest, Romania; alexandra_forray@yahoo.com; 3 Faculty of Medicine and Pharmacy, Dunarea de Jos University of Galati, 35 Al. I. Cuza Street, RO-800010 Galati, Romania; 4 Faculty of Engineering, Dunarea de Jos University of Galati, 47 Domneasca Street, RO-800008 Galati, Romania

**Keywords:** titanium implant, electrochemical impedance spectroscopy, corrosion, biological solution, reactive oxygen species

## Abstract

Pure titanium (Ti) is investigated in a pre-clinical study in Hank’s biological solution using electrochemical methods, open circuit potential, and electrochemical impedance spectroscopy to highlight the time effect in extreme body conditions, such as inflammatory diseases, on degradability due to corrosion processes occurring on the titanium implant. Electrochemical impedance spectroscopy (EIS) data are presented as Nyquist and Bode plots. The results show the increasing reactivity of titanium implants in the presence of hydrogen peroxide, which is an oxygen-reactive compound that describes inflammatory conditions. The polarization resistance, which results from electrochemical impedance spectroscopy measurements, declined dramatically from the highest value registered in Hank’s solution to smaller values registered in all solutions when different concentrations of hydrogen peroxide were tested. The EIS analysis provided insights into titanium’s in vitro corrosion behavior as an implanted biomaterial, which could not be solely obtained through potentiodynamic polarization testing.

## 1. Introduction

The interdisciplinary field of biomaterials leverages diverse sciences, such as biology, medicine, and engineering, to ensure that implants do not trigger adverse reactions when introduced into a living organism. Biomaterials interact with biological environments when implanted to restore or maintain the functionality of tissues or organs [1,2,3]. They are employed to treat, support, augment, or replace any damaged or diseased tissue, organ, or body function, thus enhancing patient health. Fundamental to any biomaterial is its biocompatibility, which is defined as the capacity of the material to interact with the host organism without triggering negative effects, like inflammation, allergies, or toxic reactions either immediately after a surgical intervention or post-operatively [2]. Moreover, a suitable biomaterial should be non-toxic, non-carcinogenic, possess requisite physical and mechanical properties, be processable into different forms, be reasonably inexpensive, and be readily available [3].

The continuous growth of the world’s population, coupled with increasing life spans, has caused the demand for biomaterials to surge, particularly metallic biomaterials. Commercial pure (CP) titanium, which was introduced for the creation of bone plates and screws in 1965, is widely used in medical implants due to its superior corrosion resistance, outstanding biocompatibility, and non-allergenic properties [4,5,6,7,8,9]. This material forms a protective oxide layer spontaneously on the implant’s surface when exposed to oxygen, providing additional benefits [10,11,12]. This oxide layer consists of titanium oxide in various oxidation states (primarily TiO_2_, Ti_2_O_3_, and TiO), and in alloys, oxidized forms of aluminum, niobium, molybdenum, or vanadium (Al_2_O_3_, Nb_2_O_5_, MoO_2_, MoO_3_ or vanadium oxides) can also be present. The layer is thermodynamically stable at physiological pH values and insoluble in biological fluids, resulting in excellent localized biocompatibility [13,14,15]. This passive electrochemical film inhibits negative ions from invading the matrix of titanium or its alloys and restricts the release of titanium or allied element ions into body fluids [16,17,18,19].

Despite titanium’s advantageous properties as an implant material, understanding how specific conditions, like inflammation, might impact its behavior is essential. Inflammation is a natural response to injury, infection, and foreign materials, leading to the release of reactive oxygen species, such as hydrogen peroxide. While the risk of titanium implant failure is low, and the body can manage the release of titanium ions into body fluids, this might not always hold true in inflammatory conditions. Inflammation could potentially disrupt titanium’s natural defenses, compromising its corrosion resistance, possibly leading to an increased release of ions into bodily fluids [20].

The high resistance of titanium to electrochemical corrosion is a well-recognized and significant property that contributes to its extensive usage in biomedical applications. This notable corrosion resistance is due to the spontaneous formation of a passive and protective titanium dioxide (TiO_2_) layer on its surface, offering a stable barrier against various aggressive environments.

Our work acknowledges this inherent quality of titanium. However, our aim extends beyond this known attribute. We aim to understand the subtle nuances in titanium’s corrosion behavior under specific conditions, notably the presence of hydrogen peroxide. Reactive oxygen species, such as H_2_O_2_, are commonly produced during inflammation—a response to implants in the human body. Therefore, it is critical to study the impact of these species on the performance of titanium implants. While titanium’s general corrosion resistance is high, the influence of inflammatory conditions and resulting reactive species on this resistance is not fully understood.

Hence, this study focuses on examining the corrosion behavior of pure titanium implants under inflammatory conditions simulated based on H_2_O_2_ presence. This insight will allow us to predict and improve the long-term stability and safety of these implants under such specific conditions.

This under-explored aspect of titanium implant behavior is the focus of our study. We aim to understand how inflammatory conditions, which are represented by reactive oxygen species, could influence the corrosion behavior of pure titanium implants. We believe that our study is a significant addition to the field, as it could help improve patient outcomes in situations where inflammation is persistent.

We use electrochemical impedance spectroscopy (EIS), which is a quantitative analysis strategy, to study the electrochemical behavior of titanium implants under various concentrations of hydrogen peroxide in a simulated biological fluid over a 48-hour immersion period [20,21,22,23]. Through an appropriate electrical circuit model fitted to the EIS results, we extracted and analyzed crucial information such as solution resistance, charge transfer resistance, bias resistance, and electrical double-layer capacitance at the electrode/electrolyte interface. We believe that our work provides new insights into the reactivity and corrosion resistance of pure titanium under inflammatory conditions, thus contributing to the design of better titanium implants and improved patient outcomes.

## 2. Results and Discussion

### 2.1. Evolution of Open Circuit Potential during One Hour after Immersion (OCP_1_)

**Figure 1:**
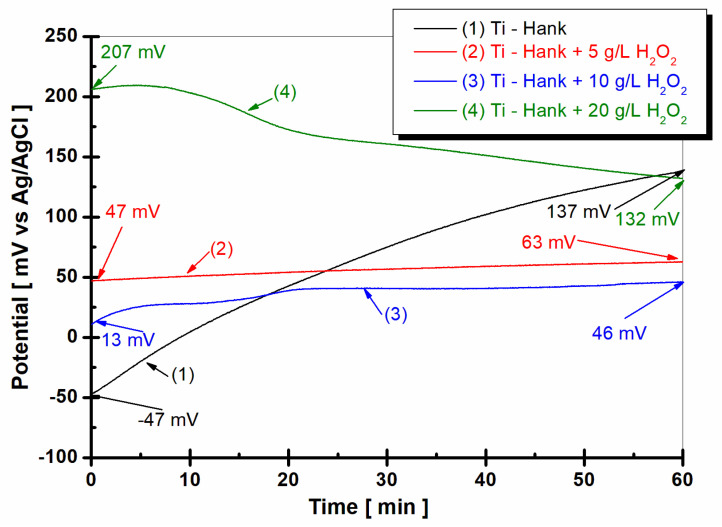
Open circuit potential of pure titanium monitored after 1 h of immersion in these working solutions: (1) Hank’s biological solution; (2) Hank solution doped with 5 g·L^−1^ H_2_O_2_; (3) Hank’s solution doped with 10 g·L^−1^ H_2_O_2_; (4) Hank’s solution doped with 20 g·L^−1^ H_2_O_2_.

The evolution of the open circuit potential of the titanium surface 1 h after immersion in the four working solutions is shown in Figure 1.

From Figure 1, it can be observed that the open circuit potential of pure titanium immersed in Hanks’ biological solution for the curve (1) started at immersion from −47 mV versus Ag/AgCl and increases slowly for one hour, reaching a more positive value of +137 mV versus Ag/AgCl. This behavior could be explained by the passivation of the titanium surface immersed in this solution.

**Figure 2:**
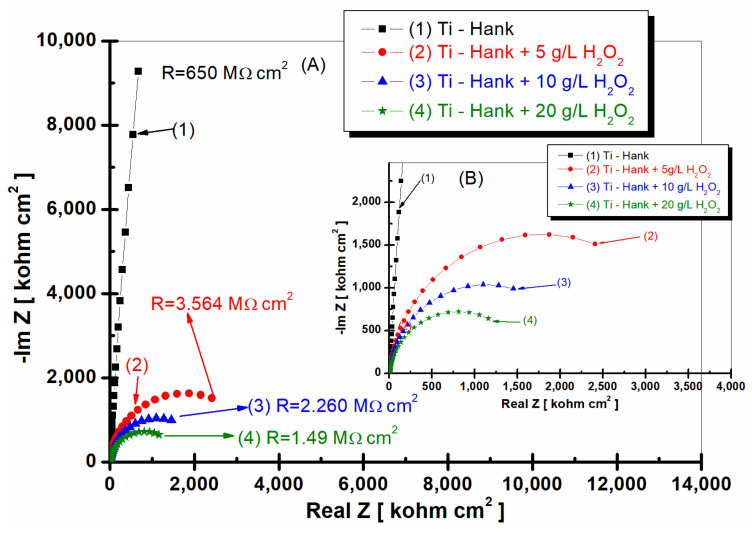
Nyquist representation of electrochemical impedance spectroscopy data recorded after 1 h of immersion of pure titanium in following working solutions: (1) Hank’s biological solution; (2) Hank’s solution doped with 5 g·L^−1^ H_2_O_2_; (3) Hank’s solution doped with 10 g·L^−1^ H_2_O_2_; (4) Hank’s solution doped with 20 g·L^−1^ H_2_O_2_. Symbols are experimental data, and a solid line is a fitting result. Layer (**A**) displays a complete Nyquist plot, while Layer (**B**) provides an amplified view of a specific region within Box A for better visual interpretation.

By adding 5 g·L^−1^ H_2_O_2_ to Hank’s solution, as shown in curve (2) in Figure 1, the open circuit potential remains almost constant from initial immersion during the entire hour of monitoring. The open circuit potential value is 47 mV versus Ag/AgCl at the beginning of immersion, which is more positive than the value obtained in Hank’s solution. After 1 h of immersion in working solution (2), the open circuit potential increases gradually to 63 mV versus Ag/AgCl. Increasing the concentration of H_2_O_2_ to 10 g·L^−1^, as shown in curve (3) in Figure 2, the value of open circuit potential decreases slowly at 13 mV versus Ag/AgCl, with the figure obtained at the immersion time being more positive than the that obtained from the Hank solution. For one hour, the value of open circuit potential shows the same trend as that observed in the working solution (2), increasing slowly to 46 mV versus Ag/AgCl. Very different behavior is observed for pure titanium immersed in Hank’s solution doped with 20 g·L^−1^ H_2_O_2_, as shown in curve (4) in Figure 1. At the immersion time, the value of open circuit potential is much more positive than the values observed for other solutions. Thus, the value of the free potential at the beginning of immersion is +207 mV versus Ag/AgCl and decreases after one hour to 132 mV, being very close to the value recorded in Hank’s solution after one hour, which is 137 mV versus Ag/AgCl.

It is well known that titanium forms a thin film of titanium oxide (TiO_2_) instantaneously in air. This film is not uniformly formed on the titanium surface; therefore, titanium could be unstable and suffer degradation due to corrosion in biological fluids. Hydrogen peroxide is a strong oxidizing compound [24,25] that is capable of reacting with titanium and native titanium dioxide (TiO_2_), potentially leading to the formation of a thicker oxide film [26,27]. The following reactions were proposed based on a combination of our observations and the recent literature [27,28]:

(1) $$\mathrm{Ti}(s)+H_{2}O_{2}(\mathrm{aq})\to {\mathrm{TiO}}_{2}(s)+H_{2}O(\mathrm{aq})$$

(2) $${\mathrm{TiO}}_{2}(s)+H_{2}O(l)\to \mathrm{TiO}{\left(\mathrm{OH}\right)}_{2}(s)$$

(3) $$\mathrm{TiO}{\left(\mathrm{OH}\right)}_{2}(s)\to {\mathrm{TiO}}_{2}(s)+S_{2}O(l)$$

(4) $${\mathrm{TiO}}_{2}(s)+H_{2}O\to \mathrm{Ti}{\left(\mathrm{OH}\right)}_{4}(s)$$

(5) $${\mathrm{TiO}}_{2}(s)+{\mathrm{OH}}^{-}(\mathrm{aq})\to {\mathrm{TiO}}_{2}{\left(\mathrm{OH}\right)}^{-}(\mathrm{aq})$$

(6) $$\mathrm{Ti}{\left(\mathrm{OH}\right)}_{4}(s)\to {\mathrm{TiO}}_{2}(s)+2H_{2}O(l)$$

Titanium could react with hydrogen peroxide (H_2_O_2_), forming a titanium oxide film (Equation (1)). Furthermore, some of the titanium dioxide (TiO_2_) could be hydrolyzed (Equations (2) and (3)). The reaction of hydration (Equation (4)) occurs simultaneously with the formation of Ti–OH functional groups. The formation of Ti–OH functional groups (Equation (5)) could contribute to reducing the open circuit potential to a more negative value during the immersion period, followed by a continuous shift to more positive values. This behavior aligns with the EIS results presented later on in the manuscript. Hydrated and hydrolyzed titanium dioxide (TiO_2_) may lead to the creation of negatively charged surfaces (Equations (5) and (6)), which could play a role in reducing the open circuit potential to a more negative value during the immersion period, followed by a continuous shift to more positive values, as explained in Equation (1). This behavior is also supported by the EIS results presented later on in the manuscript.

The passive film on pure titanium is usually associated with TiO_2_, Ti_2_O_3_, and TiOOH groups. In the presence of H_2_O_2_, the TiO_2_/Ti_2_O_3_ couple could undergo dismutation, as described in the following reactions [27,29]

(7) $$2{\mathrm{TiO}}_{2}(s)+H_{2}O_{2}(\mathrm{aq})\to {\mathrm{Ti}}_{2}O_{3}(s)+H_{2}O(l)+O_{2}(g)$$

(8) $${\mathrm{Ti}}_{2}O_{3}(s)+H_{2}O_{2}(\mathrm{aq})\to 2\mathrm{TiOOH}(s)+H_{2}O(l)$$

The reactions proposed could account for the observed behavior in open circuit potential monitoring.

The intricate reactions taking place on the surface of pure titanium in biological solutions under extreme inflammatory conditions could account for the observed behavior in open circuit potential monitoring.

Similar results with more positive values of OCP were obtained immediately after immersion in PBS solution samples with increasing H_2_O_2_ concentrations by studying the titanium alloy Ti6Al4V [24,28,30,31,32]

### 2.2. Electrochemical Impedance Spectroscopy after One Hour from Immersion (EIS_1_)

**Figure 3:**
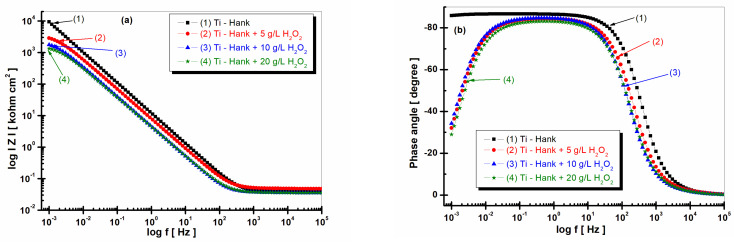
Bode plots of electrochemical impedance spectroscopy data recorded after 1 h of immersion of pure titanium in following working solutions: (1) Hank’s biological solution; (2) Hank’s solution doped with 5 g·L^−1^ H_2_O_2_; (3) Hank’s solution doped with 10 g·L^−1^ H_2_O_2_; (4) Hank’s solution doped with 20 g·L^−1^ H_2_O_2_. (**a**) Module Z vs. logf; (**b**) phase angle vs. logf. Symbols are experimental data, and solid line is fitting result.

The first measurement of EIS was performed consecutively after the first OCP, i.e., after 1 h of immersion, to free potential. The EIS diagrams were, thus, obtained for each test solution in the first hour after immersion, as can be seen in Figure 2 (Nyquist plots) and Figure 3, (Bode plots).

The studied interfaces between pure titanium as a solid electrode and working biological solutions show a non-capacitive behavior with a frequency dispersion; thus, the proposed experimental data modeling system has a constant phase element instead of an electric double-layer capacity. The impedance of such a system is described in the following equation [24]:

(9) $$Z_{\mathrm{CPE}}=\frac{1}{{Q(j\omega )}^{\alpha }}$$

where α is the CPE exponent that varied between 0 and 1, ω is an angular frequency (ω = 2πf), and one of $j=\sqrt{-1}$ and Q measured in Fs^−(1−α)^ or Ω^−1^s^α^ is the CPE parameter. Interfacial capacitance is usually determined based on the value of Q when α → 1, though this approach is inaccurate [33].

Theoretical and experimental works are carried out to understand the origin of CPE behavior, which is still controversial. In general, the behavior is attributed to the inhomogeneity of the studied surfaces, roughness, and porosity, as well as to the adsorption of specific anions [33].

**Figure 4:**
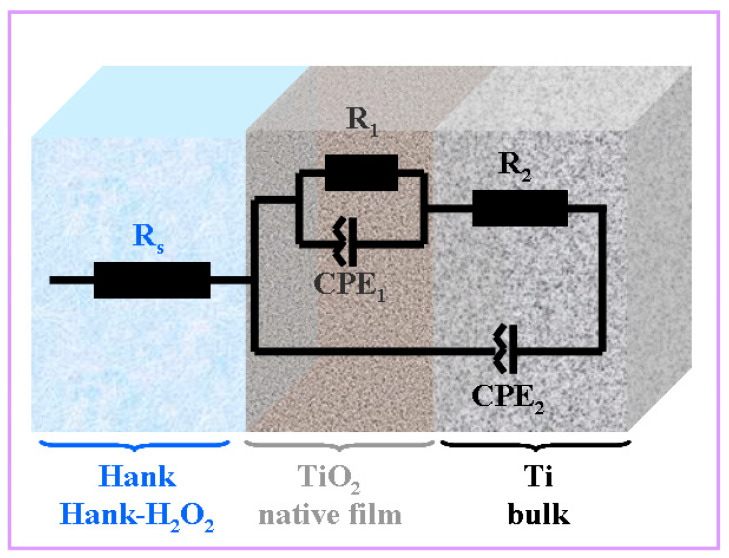
Equivalent electrical circuit used for impedance experimental data fitting of pure titanium immersed in Hank’s solution and Hank’s solution doped with different H_2_O_2_ concentrations. R_s_ is the solution resistance; R_1_-CPE_1_ are titanium oxide native film resistance and constant phase element, respectively; R_2_-CPE_2_ are bulk titanium resistance and constant phase element, respectively.

The experimental data obtained were fitted using the Z_view_ software, with the correctness of the fitting being given by the Chi-Squared parameter with a value of 10^−4^. Thus, the equivalent circuit that gave the best fits is presented in Figure 4.

As can be seen in Figure 2 and Figure 3, pure titanium exhibits good corrosion resistance and weak reactivity in Hank’s biological solution for curve (1), resulting in a specific resistance of 650 Mohm·cm^2^.

By adding 5 g·L^−1^ H_2_O_2_ to Hank’s solution, the specific resistance of pure titanium decreases to 3.564 Mohm·cm^2^.

Increasing the concentration of H_2_O_2_ to 10 and 20 g·L^−1^ further leads to a decrease in the specific resistance of pure titanium immersed in the two solutions. Thus, at the concentration of 10 gL^−1^ of reactive oxygen species, the specific resistance of pure titanium shows a value of 2260 Mohm·cm^2^, as can be seen in curve (2) of Figure 2, while at 20 g·L^−1^ of reactive oxygen species, the specific resistance drops to the value of 1.49 Mohm·cm^2^, as shown in curve (4) of Figure 2. 

This decrease in the specific resistance of pure titanium in the Hank’s solution doped with increased concentrations of the inflammatory compound can be explained based on the prevalence of Equations (1) and (7), with the titanium oxide film becoming unstable through its dissolution by H_2_O_2_, which leads to the increase in titanium reactivity in these inflammatory conditions.

Similar results were observed by other authors [28] who studied the reactions of the inflammatory compound H_2_O_2_ in PBS solution with the titanium alloy Ti6Al4V. They observed the increase in the corrosion of titanium in correlation with the increase in the concentration of H_2_O_2_ (from 0.8 mM to 330 mM), suggesting the formation of a Ti-H_2_O_2_ complex that further led to the formation of a hydrated titanium compound: Ti(OOH)_n_ [28,34].

The impedance modulus of pure titanium in biological Hank’s solution, as shown in Figure 3a, is about 10 times higher than the impedance modulus resulting from other working solutions, as shown in curve (1). The phase angle of pure titanium in Hank’s solution shows a constant high value (>–80 degrees) on a large frequency domain, as shown in Figure 3b curve (1), compared to the phase angle exhibited in Hank’s solution doped with different concentrations of H_2_O_2_, as shown in curves (2, 3, 4) in Figure 3b, which show an increasing value of about –80 degree in a narrow frequency range, before decreasing further to −30 degree.

### 2.3. Monitoring the Open Circuit Potential during 12 h after 36 h from Immersion (OCP_2_)

**Figure 5:**
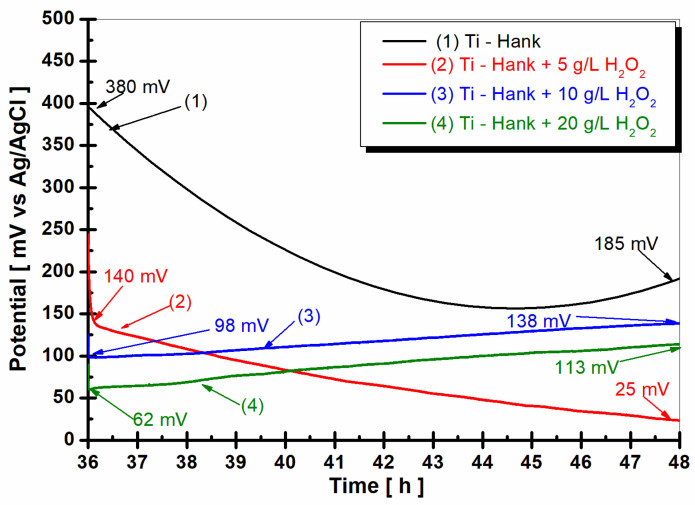
Open circuit potential of pure titanium monitored for 12 h, after 36 h from the immersion in the working solutions: (1) Hank’s biological solution; (2) Hank’s solution doped with 5 g·L^−1^ H_2_O_2_; (3) Hank’s solution doped with 10 g·L^−1^ H_2_O_2_; (4) Hank’s solution doped with 20 g·L^−1^ H_2_O_2_.

The open circuit potential (OCP_2_) measured after 36 h of immersion and 12 h of pure titanium immersion in all working solutions is shown in Figure 5.

In the Hank’s biological solution, pure titanium shows a tendency toward more positive values, suggesting good stability. However, the presence of 5 g·L^−1^ H_2_O_2_ seems to shift the open circuit potential towards more negative values. Higher concentrations of H_2_O_2_ (10 g·L^−1^ and 20 g·L^−1^) exhibit unique stability, which is indicative of complex reactions involving titanium and reactive oxygen species under inflammatory conditions. This complexity could increase the reactivity of pure titanium as an implant, which is supported by the dynamic Equations (1)–(8), which are related to titanium and titanium dioxide dissolution and passive film formation.

### 2.4. Electrochemical Impedance Spectroscopy after 48 h from Immersion (EIS_2_)

**Figure 6:**
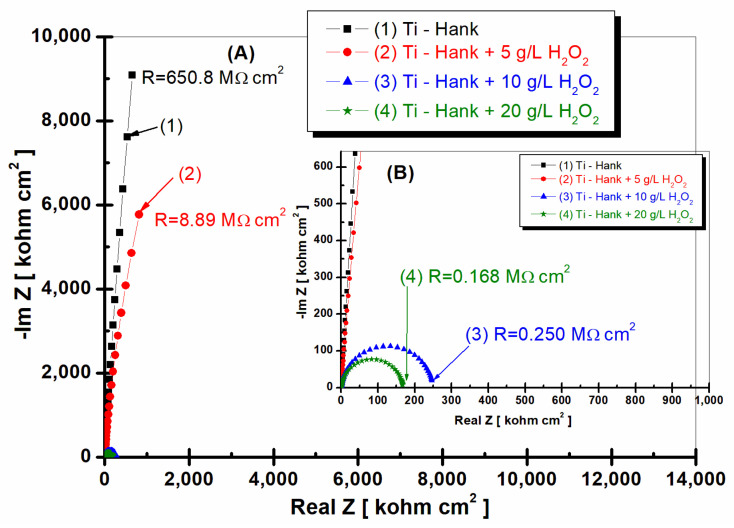
Nyquist representation of electrochemical impedance spectroscopy data recorded after 48-hour immersion of pure titanium in these working solutions: (1) Hank’s biological solution; (2) Hank’s solution doped with 5 g·L^−1^ H_2_O_2_; (3) Hank’s solution doped with 10 g·L^−1^ H_2_O_2_; (4) Hank’s solution doped with 20 g·L^−1^ H_2_O_2_. Symbols are experimental data, and solid line is fitting result. Layer (**A**) displays complete Nyquist plot, while Layer (**B**) provides an amplified view of a specific region within Box A for better visual interpretation.

**Figure 7:**
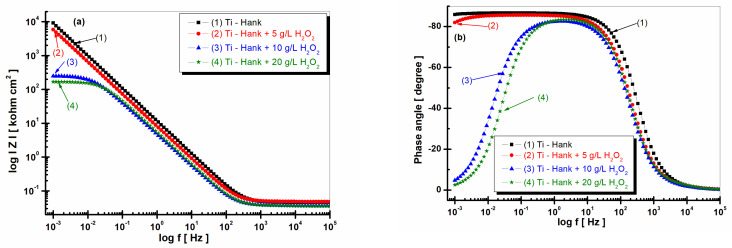
Bode plots of electrochemical impedance spectroscopy data recorded after 48-hour immersion of pure titanium in these working solutions: (1) Hank’s biological solution; (2) Hank’s solution doped with 5 g·L^−1^ H_2_O_2_; (3) Hank’s solution doped with 10 g·L^−1^ H_2_O_2_; (4) Hank’s solution doped with 20 g·L^−1^ H_2_O_2_. (**a**) Module Z vs. logf; (**b**) Phase angle vs. logf. Symbols are experimental data, and solid line is fitting result.

Figure 6 and Figure 7 display the EIS2 experimental data (symbol markers) and fitting outcomes (solid line) after 48 h of immersion, which are presented in the Nyquist (Figure 6) and Bode plots (Figure 7a,b).

Pure titanium demonstrates commendable stability in Hank’s biological solutions after 48 h of immersion (curve 1, Figure 6 and Figure 7), maintaining a high specific resistance akin to the value recorded one hour post-immersion, which was 650.8 Mohm·cm^2^.

Upon the addition of 5 g·L^−1^ H_2_O_2_ to Hank’s solution, pure titanium’s specific resistance gradually increases to 8.89 Mohm·cm^2^, marking an increase compared to the resistance demonstrated 1 h post-immersion (curve 2, Figure 6). This result suggests that at this H_2_O_2_ concentration, pure titanium’s reactivity could diminish over time, possibly due to the self-reparation of the protective oxide film on the material’s surface.

In contrast, when the H_2_O_2_ concentration escalates to 10 and 20 g·L^−1^, the specific resistance of pure titanium decreases roughly tenfold after 48 h of immersion compared to values recorded 1 h after immersion (curves 3 and 4, Figure 6). Specifically, at 10 g·L^−1^ H_2_O_2_, the specific resistance plummets from an initial 2.26 Mohm·cm^2^ to 0.25 Mohm·cm^2^ (curve 3, Figure 2 and Figure 6). The same behavior is observed at a concentration of 20 g·L^−1^ H_2_O_2_, where the specific resistance of pure titanium decreases from an initial value of 1.49 Mohm·cm^2^ to 0.168 Mohm·cm^2^ after 48 h of immersion (curves 4, Figure 2 and Figure 6).

The impedance modulus of pure titanium immersed in Hank’s biological solution, as shown in Figure 7a curve (1), is sustained at high values, even after 48 h of immersion. Remarkably, the impedance modulus of pure titanium immersed in Hank’s solution augmented with 5 g·L^−1^ H_2_O_2_ increases after 48 h, corresponding to the values achieved for pure titanium in undoped Hank’s solution (curve 2, Figure 7a). This observation offers intriguing insights into titanium’s dynamic electrochemical responses under varied conditions.

**Figure 8:**
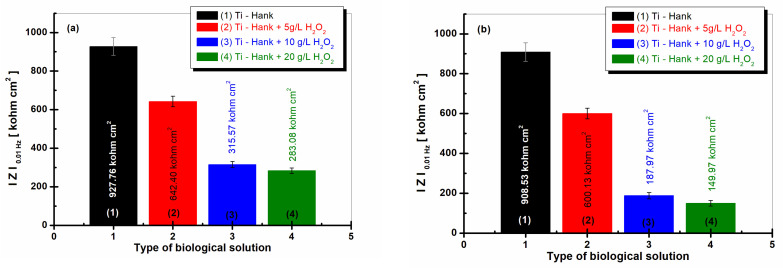
Low-frequency impedance modulus (at 0.01 Hz) for pure Ti immersed in different biological solutions: (1) Hank’s biological solution; (2) Hank’s solution doped with 5 g·L^−1^ H_2_O_2_; (3) Hank’s solution doped with 10 g·L^−1^ H_2_O_2_; (4) Hank’s solution doped with 20 g·L^−1^ H_2_O_2_. (**a**) After 1 h of immersion; (**b**) after 48 h of immersion.

For pure titanium immersed in working solutions containing 10 and 20 g·L^−1^ of reactive oxygen species, the impedance modulus after 48 h of immersion exhibits lower values relative to those documented after 1 h of immersion (curves 3 and 4, Figure 8a).

The phase angle of pure titanium in Hank’s solution after 48 h of immersion shows a constant high value (>80 degrees) on a large frequency domain, as shown by Figure 7b’s curve (1). The phase angle of pure titanium immersed in Hank’s solution doped with 5 g·L^−1^ H_2_O_2_ after 48 h of immersion is approaching the values recorded for Hank’s solution without H_2_O_2_ using a large frequency domain, as shown by curve (2) in Figure 7b. Only at a lower frequency did the phase angle in this solution tend to record lower values compared to those obtained in the undoped Hank solution.

The phase angle of pure titanium, which was revealed after 48 h of immersion, in the other two solutions with 10 and 20 g·L^−1^ H_2_O_2_, respectively, shows a value below −80 degrees in a narrow frequency range, before dropping much more rapidly after the first hour of immersion, reaching approximately −4 degrees at low frequencies.

This behavior confirms the increase in titanium reactivity and, therefore, the decrease in corrosion resistance when the hydrogen peroxide inflammatory compound has a higher concentration, respectively, at 10 or 20 g·L^−1^ H_2_O_2_.

The results follow those of other authors who studied the effect of hydrogen peroxide species present in PBS solution on Ti6Al4V alloy [26].

### 2.5. Impedance Modulus at Low Frequency, ${\left|Z\right|}_{0.01\mathrm{Hz}}$

The impedance modulus at low frequency can be correlated with the processes that take place at the interface of titanium with the solution (electrolyte), i.e., where the corrosion phenomena start to occur [26,34]; therefore, it is an effective tool for the elucidation of the studied electrode/electrolyte systems.

The values of the low-frequency impedance modulus, ${\left|Z\right|}_{0.01\mathrm{Hz}}$, are presented in Figure 8a and were obtained after 1 h of immersion, while the values in Figure 8b were obtained after 48 h of immersion.

The impedance modulus at 0.01 Hz of pure titanium is highest in Hank’s biological solution after 1 h of immersion, as well as after 48 h of immersion, with both measures having similar valued of 927.86 Kohm·cm^2^ and 908.53 Kohm·cm^2^, respectively, confirming the good stability and low reactivity of this biological solution.

For the Hank’s solution doped with 5 g·L^−1^ H_2_O_2_, the impedance modulus at low frequency, ${\left|Z\right|}_{0.01\mathrm{Hz}}$, is 642.40 Kohm·cm^2^ after 1 h of immersion, while column (2) from Figure 8a shows a similar value after 48 h of immersion, i.e., 600.13 Kohm·cm^2^, as shown in column (2).

For the other two working solutions with 10 and 20 g·L^−1^ H_2_O_2_, the impedance modulus, ${\left|Z\right|}_{0.01\mathrm{Hz}}$, is lower after the first hour of immersion, having values of 315.57 Kohm·cm^2^ and 283.08 Kohm·cm^2^, respectively, in curves (3) and (4) from Figure 8a. These values decrease significantly after 48 h of immersion, with values of 187.87 Kohm·cm^2^ and 149.97 Kohm·cm^2^, respectively, recorded in curves (3) and (4) from Figure 8b.

### 2.6. Potentiodynamic Polarization Diagrams

**Figure 9:**
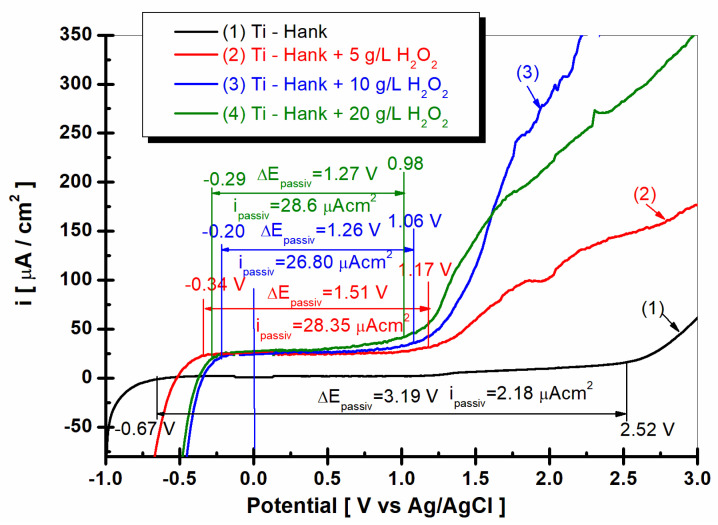
Potentiodynamic polarization diagrams (PD) were recorded in potential domain from −1 V to 3 V vs. Ag/AgCl, with a scan rate of 1 mV/s in these working solutions: (1) Hank’s biological solution; (2) Hank’s solution doped with 5 g·L^−1^ H_2_O_2_; (3) Hank’s solution doped with 10 g·L^−1^ H_2_O_2_; (4) Hank’s solution doped with 20 g·L^−1^ H_2_O_2_.

To investigate the effect of the inflammatory compound on the passive domain of pure titanium immersed in Hank’s solution, the potentiodynamic polarization curves were plotted, the results of which are shown in Figure 9.

As shown in Figure 9, curve (1) pure titanium immersed in Hank’s solution shows a wide range of passivation between E_1_ = −068 V versus Ag/AgCl and E_2_ = 2.52 V versus Ag/AgCl, having a passivation domain of ΔE_passiv_ = 3.19 V. The average passivation current density is low, being i_passiv_ = 2.18 μA·cm^−2^ for curve (1). 

**Figure 10:**
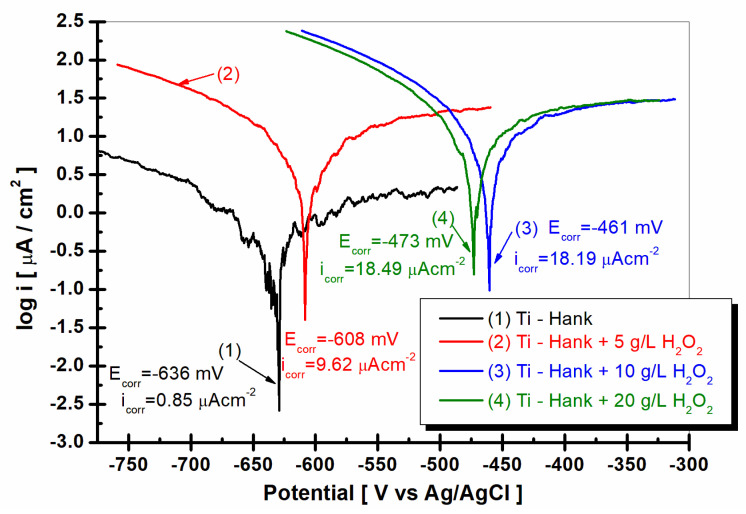
Tafel plots as log i versus E, recorded with a scan rate of 1 mV/s in these working solutions: (1) Hank’s biological solution; (2) Hank’s solution doped with 5 g·L^−1^ H_2_O_2_; (3) Hank’s solution doped with 10 g·L^−1^ H_2_O_2_; (4) Hank’s solution doped with 20 g·L^−1^ H_2_O_2_.

The passivation domains of pure titanium become narrower when immersed in Hank’s solution with different concentrations of the inflammatory compound, i.e., H_2_O_2_. Thus, in the Hank’s solution doped with 5 g·L^−1^ H_2_O_2_, the passivation domain of pure titanium manifests itself between E_1_ = −0.34 V versus Ag/AgCl and E_2_ = 1.17 V versus Ag/AgCl, with a passive domain of ΔE_passiv_ = 1.51 V, which is narrower than that observed in the undoped Hank’s solution. The passivation current density increases to i_passiv_ = 28.35 μAcm^−2^, as shown by curve (2) in Figure 10.

For the Hank’s solution doped with 10 g/L H_2_O_2_, the passive domain of pure titanium becomes even narrower, appearing between E_1_ = −0.2 and E_2_ = 1.06 V versus Ag/AgCl, with the passivation domain being ΔE_passiv_ = 1.26 V, while the average of passivation current density is greater, being i_passiv_ = 26.80 μA·cm^−2^.

The passive domain of pure titanium immersed in Hank’s solution doped with 20 g·L^−1^ H_2_O_2_ stretches between the potentials E_1_ = −0.29 V and E_2_ = 0.98 V versus Ag/AgCl, and the passivation current density is ΔE_passiv_ = 1.27 V. 

The average passivation current density continues to increase at i_passiv_ = 28.6 μA·cm^−2^.

The drastic narrowing of the passivation domain of pure titanium immersed in Hank’s solution in the presence of the inflammatory compound, as well as the increase in the passivation current density, confirms, once again, the increase in the reactivity of pure titanium under these conditions; at the same time, it confirms the decrease in corrosion resistance through the dissolution of both titanium and the titanium oxide film on its surface through the reactions described in Equations (1)–(8).

### 2.7. Linear Polarization and Tafel Extrapolation Plots

To complete the results regarding the degradation of the implant material in inflammatory conditions, Tafel extrapolation from the linear polarization curves was performed. Thus, the corrosion current density of titanium in the studied working solutions can be compared, as can be seen in Figure 10.

As can be seen in Figure 10, the corrosion current density for titanium immersed in Hank solution is 0.85 μA·cm^−2^. For all other solutions with added hydrogen peroxide, the corrosion current density increases at 9.62 μA·cm^−2^ for 5 g·L^−1^ H_2_O_2_, at 18.19 μA·cm^−2^ for 10 g·L^−1^ H_2_O_2_, and at 18.49 μA·cm^−2^ for 20 g·L^−1^ H_2_O_2_. The corrosion rate of titanium, which is expressed in μm·y^−1^, increases from 9.2 for Hank’s solution to 136.0 for Hank’s solution doped with 5 g·L^−1^ H_2_O_2_, 266.6 for Hank’s solution doped with 10 g·L^−1^ H_2_O_2_, and 281.2 for Hank’s solution doped with the highest concentration of H_2_O_2_ (20 g·L^−1^). The corrosion rates resulting from Tafel plots are in agreement with specific resistances resulting from electrochemical impedance spectroscopy data. The titanium implant is susceptible to higher degradation due to inflammatory conditions.

### 2.8. Optical Microscopy before and after Corrosion

**Figure 11:**
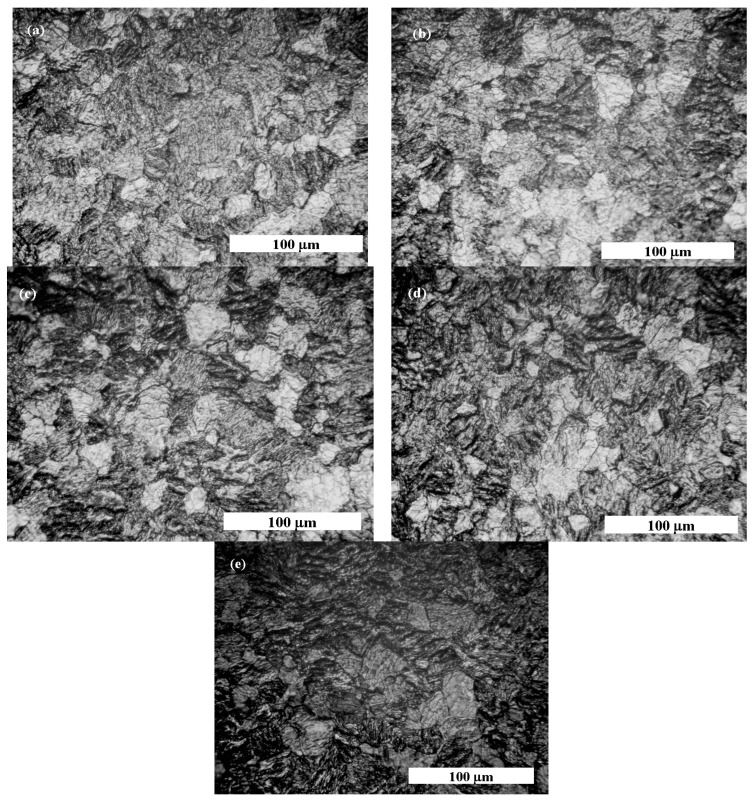
Optical microscopy morphology of pure titanium obtained at following stages: (**a**) before corrosion tests; (**b**) when pure titanium is immersed in Hank’s solution; (**c**) when pure titanium is immersed 48 h in Hank’s solution and 5 g·L^−1^ H_2_O_2_; (**d**) when pure titanium is immersed 48 h in Hank’s solution and 10 g·L^−1^ H_2_O_2_; (**e**) when pure titanium is immersed 48 h in Hank’s solution and 20 g·L^−1^ H_2_O_2_.

As is presented in Materials and Methods, the samples of pure titanium ere investigated for surface morphology before and after immersion in working solutions for 48 h without any other measurement. The results are presented in Figure 11a–e.

The pure titanium’s surface before corrosion tests shows a uniform surface covered with titanium oxide passive film, as highlighted by light grey zones in Figure 11a. After immersion in Hank’s biological solution, pure titanium has more light grey zones, as shown in Figure 10b, due to passivation of its surface in the biological solution without an inflammatory compound. 

By adding 5 g·L^−1^ of H_2_O_2_ to Hank’s solution, the surface morphology of pure titanium shows more light grey areas, such as islands surrounded by darker areas that can be assimilated into the titanium corrosion process through which the passive film dissolved, as shown in Figure 11c. Regarding the morphology shown in Figure 11d, the darker areas became wider, while the light gray areas became less wide, which shows that pure titanium immersed in Hank’s solution doped with 10 g·L^−1^ H_2_O_2_ loses much of the oxide film on its surface, becoming more prone to dissolution and, therefore, the corrosion process. The most affected surface of pure titanium is observed for the morphology shown in Figure 11e, which was immersed in the Hank’s solution doped with the highest concentration of H_2_O_2_. It was observed that the surface of titanium became almost entirely darker with very small areas of undissolved light grey oxide film.

The surface morphology study of pure titanium immersed in the working solutions confirms the electrochemical results obtained via impedance spectroscopy measurements.

## 3. Materials and Methods

Commercially available pure titanium (Ti) was purchased from Goodfellow SARL, Lille, France, and had a thickness of 3 mm and dimensions of 300 × 300; the titanium was then cut into pieces of 25 × 25 mm in size. An insulated copper wire was attached to each sample outside of contact with the sample. After attaching the wire to ensure electrical contact and connection with the electrochemical workstation, each sample was protected on the opposite side and the edges with insulating resin to ensure a measurable and constant active surface of 3 cm^2^.

Before corrosion investigations, the samples were cleaned with 0.5 M of sodium hydroxide, rinsed well with distilled water, and dried.

The electrochemical tests of titanium behavior were performed in the artificial body fluid known as Hank’s solution. 

Hank’s solution, which was developed to mimic the ionic environment of human blood plasma, is frequently used in biomedical studies involving cells and tissues. It is important to note that while Hank’s solution did not replicate all of the complexity of the physiological environment, it provided a reasonably accurate approximation for in vitro studies.

The composition of Hank’s solution is shown in Table 1 [23].

Hydrogen peroxide, which is present in body fluids as an inflammatory compound, was added in certain amounts to Hank’s solution to simulate a local inflammatory environment. The chosen concentrations of H_2_O_2_ (5 g/L, 10 g/L, and 20 g/L) were reflective of the increasing severity of inflammation. These specific concentrations were selected based on the work of Prestat and Thierry (2021), who demonstrated that these ranges are representative of physiological inflammation conditions. By testing these varying concentrations, we aimed to better understand how different degrees of inflammation might influence the corrosion behavior of titanium implants. It should be noted that during inflammation, local pH levels can decrease significantly due to factors like increased metabolic activity and the accumulation of reactive oxygen species, such as H_2_O_2_. This problem can affect the corrosion behavior of implants, and we aimed to simulate this effect in our study. Consequently, we did not use a buffer to maintain the pH at a constant physiological level. The physicochemical characteristics of the working solutions used are presented in Table 2.

To evaluate and monitor the corrosion resistance of pure titanium when placed in the working solutions specified in Table 2, an electrochemical cell with three electrodes connected to an electrochemical workstation was used, along with a solution volume constant at 150 mL. The working electrode (WE) consisted of pure titanium samples (Ti) prepared as specified above. The counter electrode (CE) was a platinum mesh with an active surface of 3 cm^2^, while the reference electrode (RE) was Ag/AgCl placed in saturated KCl solution, with a constant potential of +199 mV versus the normal hydrogen electrode (NHE). All experiments were carried out at 37 ± 0.5 °C and repeated three times to check reproducibility.

**Figure 12:**
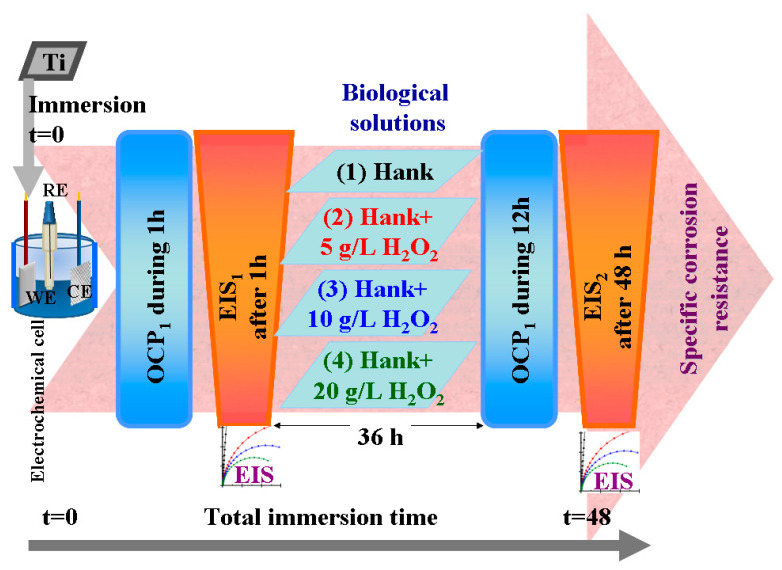
Schematics of electrochemical setup and protocol used to evaluate corrosion behavior of pure titanium in working Hank’s solutions and Hank’s doped with different amounts of hydrogen peroxide to create inflammatory conditions.

For each working solution, after immersing the sample in the electrochemical cell and connecting it to the electrochemical workstation, the sample was applied to the electrochemical protocol of measurements shown in Figure 12, which consisted of the following steps:

- Measuring the open circuit potential (OCP_1_) for one hour until being sure that we had arrived at a steady state of titanium surface in the tested solution;
- Measuring the electrochemical impedance spectroscopy (EIS_1_) at free potential with an amplitude of sinusoidal potential of 10 mA, ranging from a high frequency of 100 kHz to a low frequency of 1 mHz, with 10 frequencies per decade;
- Leaving the samples immersed in the studied solutions for 36 h and resuming the cycle of open circuit potential and electrochemical impedance spectroscopy measurements.
- Measuring the open circuit potential (OCP_2_) for 12 h to evaluate the surface stability after a long immersion time (36 h).
- Measuring the electrochemical impedance spectroscopy (EIS_2_) at free potential with an amplitude of sinusoidal potential of 10 mA, ranging from a high frequency of 100 kHz to a low frequency of 1 mHz, with 10 frequencies per decade. This measurement gives us information about any changes and reactivity occurring on the titanium surface after 48 h in the studied solutions.

Separately, for other samples, the passivation range of pure titanium in the specified solutions was studied to observe its effect on the presence of reactive oxygen species. Thus, the potentiodynamic polarization curves were measured in the potential range from −1 V versus Ag/AgCl to +3 V versus Ag/AgCl, with the scan rate of the potential set at 1 mV·s^−1^.

Linear polarization and Tafel plot extrapolations were performed in order to compare the corrosion current density and the corrosion rates (μm·y^−1^) in the tested working solutions.

The surface morphology of pure titanium was examined before and after a 48-hour period of immersion in the working solutions using an Optika IM5 optical microscope.

## 4. Conclusions

The key findings of this research can be outlined as follows. 

Pure titanium exhibits good corrosion resistance and weak reactivity in Hank’s biological solution. However, the presence of H_2_O_2_, which is a common inflammatory compound, in the solution leads to increased reactivity and a decrease in corrosion resistance, as evidenced by the decrease in specific resistance and low-frequency impedance modulus with increasing H_2_O_2_ concentration.

The open circuit potential measurements reveal increased reactivity of pure titanium under inflammatory conditions, which can be attributed to the dynamic dissolution of titanium and titanium dioxide and the continuous formation of the titanium dioxide passive film.

Electrochemical impedance spectroscopy and low-frequency impedance modulus measurements confirm the impact of inflammatory conditions given by H_2_O_2_ on the corrosion behavior of pure titanium. The impedance modulus at 0.01 Hz is highest for pure titanium in Hank’s biological solution without H_2_O_2_, both after 1 and 48 h of immersion. The smallest impedance modulus at a low frequency is recorded for the highest concentrations of inflammatory reactive oxygen species. 

The passivation potential domain of titanium in biological solution is affected by inflammatory reactive species becoming narrower with increasing passive current densities. 

The corrosion rates calculated via Tafel plot extrapolation confirm the electrochemical spectroscopy results, as the values increase when Hank’s solution is replaced with Hank’s doped solution for different concentration of H_2_O_2_.

Optical microscopy reveals that the surface morphology of pure titanium changes when immersed in working solutions containing H_2_O_2_. The titanium oxide passive film dissolves, leading to darker areas on the surface and increased susceptibility to corrosion.

The concentration aspect of H_2_O_2_ throughout the experiments could be a focus for future research in order to assess how the concentration of H_2_O_2_ changes over time in the experimental setup, as well as how these changes may impact the corrosion behavior of pure titanium.

Given these findings, it is crucial to point out that while our research focuses on pure titanium, understanding its reactivity and corrosion resistance under inflammatory conditions provides a baseline for future studies. Comparative research involving other implant materials, such as Ti-6Al-4V, Co-Cr, and Co-Cr-Mo alloy, under similar conditions would augment this study and contribute to a comprehensive understanding of implant performance in inflammatory conditions. The present research could serve as a stepping stone for launching such comparative studies.

Overall, the study highlights the importance of understanding the corrosion behavior of titanium implants under various physiological conditions, including those involving inflammation and reactive oxygen species. The presence of H_2_O_2_ significantly impacts the corrosion resistance of pure titanium, which could have implications for the performance and longevity of titanium-based implants in the human body. This knowledge could also guide further research on improving the corrosion resistance of titanium implants and optimizing their performance under inflammatory conditions.

## Figures and Tables

**Table 1:** Composition of biological Hank’s solution.

Nr. Crt.	Chemical Compound	Concentration g/L
1	NaCl	8.8
2	KCl	0.4
3	CaCl_2_·2H_2_O	0.35
4	Na_2_HPO_4_·H_2_O	0.25
5	MgCl_2_	0.19
6	MgSO_4_·7H_2_O	0.06
7	C_6_H_12_O_6_	1

**Table 2:** Physicochemical characteristics of biological Hank’s solution and modified Hank’s solution with hydrogen peroxide.

Nr. Crt.	Solution Type	pH	Conductivity [mS/cm]	Salinity [ppt]
1	Hank	7.4 ± 0.5	15.4 ± 0.1	8.9 ± 0.1
2	Hank and 5 g/L H_2_O_2_	6.55 ± 0.2	14.01 ± 0.2	8.2 ± 0.1
3	Hank and 10 g/L H_2_O_2_	6.47 ± 0.3	14.2 ± 0.1	8.2 ± 0.1
4	Hank and 20 g/L H_2_O_2_	6.32 ± 0.2	13.9 ± 0.1	8.0 ± 0.1

## Data Availability

No data availability due to due to privacy to publish the results.
